# 2941. Racial and Ethnic Disparities in the Care Cascade for Children with Perinatal Hepatitis C Virus (HCV) infection in the United States (US)

**DOI:** 10.1093/ofid/ofad500.180

**Published:** 2023-11-27

**Authors:** Megan Rose Curtis, Ben Buzzee, Breanne E Biondi, Benjamin P Linas, Andrea L Ciaranello, Rachel L Epstein

**Affiliations:** Brigham and Women's Hospital, Massachusetts General Hospital, Harvard Medical School, Boston Medical Center, Boston, MA; Boston Medical Center, Boston, Massachusetts; Boston University School of Public Health, Boston, Massachusetts; Boston University School of Medicine/Boston Medical Center, Boston, MA; Massachusetts General Hospital, Boston, Massachusetts; Boston University Chobanian and Avedisian School of Medicine, Boston, Massachusetts

## Abstract

**Background:**

From 2000-2019 HCV prevalence among pregnant people increased 10-fold in the US, in parallel to the opioid epidemic, with consequences for children, for whom perinatal transmission is the most common route of infection. Direct-acting antivirals (DAAs) were first approved for children ≥3 years old in 2019. The aim of this study was to characterize the care cascade among children with perinatal HCV and to examine racial and ethnic disparities in access to HCV care.

**Methods:**

Analyzing 2014-2022 data from TriNetX, a research network of electronic health record data including 90 million individuals within US healthcare organizations, we defined a cohort of children born between 2010-2019 with a positive HCV ribonucleic acid (RNA) test. We defined linkage as: 1) HCV genotype test, 2) encounter with HCV as primary diagnosis, or 3) liver fibrosis testing. We tabulated the number of children prescribed DAAs and tested for sustained virologic response 12 weeks post-treatment (SVR12). We used logistic regression models to estimate odds of linkage and of receiving DAAs across racial and ethnic groups.

**Results:**

Among 766 HCV RNA positive children, 211 (28%) linked to care. Only 40 children (5%; 19% of those linked) were prescribed DAAs and 15 (38% of those treated) had SVR12 assessed, all of whom achieved cure. Black and Hispanic children had 62% and 70% odds of linking (OR 0.38, 95%CI 0.19-0.76 and OR 0.30, 95%CI 0.18-0.51) when compared to White and non-Hispanic and children, respectively. Hispanic children had 72% lower odds of receiving DAAs compared with non-Hispanic children (OR 0.28, 95%CI 0.08-0.94). Disparities persisted for linkage when adjusted by region for Black and Hispanic children (OR 0.38, 95% CI 0.19-0.78 and OR 0.34, 95% CI 0.20-0.59) when compared to White and non-Hispanic children, respectively.

**Figure:**
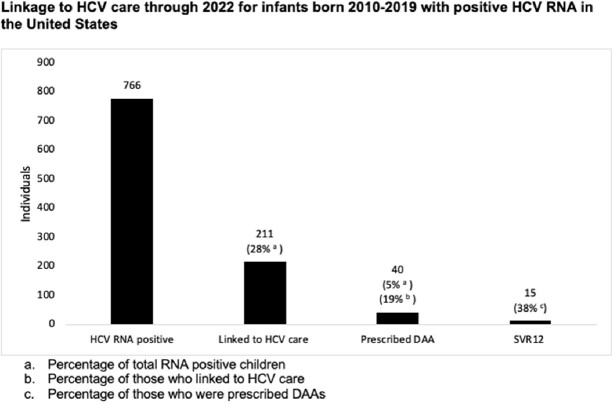

**Conclusion:**

Despite DAA approvals for children ≥3 years old, most children with HCV are not yet linked or treated. This is a missed opportunity to prevent later health complications. Our study is the first to characterize the HCV care cascade in a large national cohort of children eligible for DAAs. We reveal major disparities in HCV care access for children, highlighting how infectious complications of the opioid epidemic might drive intergenerational disparities in health.

**Disclosures:**

**All Authors**: No reported disclosures

